# Expanded phenotypic spectrum of neurodevelopmental and neurodegenerative disorder Bryant-Li-Bhoj syndrome with 38 additional individuals

**DOI:** 10.1038/s41431-024-01610-1

**Published:** 2024-04-27

**Authors:** Dana E. Layo-Carris, Emily E. Lubin, Annabel K. Sangree, Kelly J. Clark, Emily L. Durham, Elizabeth M. Gonzalez, Sarina Smith, Rajesh Angireddy, Xiao Min Wang, Erin Weiss, Annick Toutain, Roberto Mendoza-Londono, Lucie Dupuis, Nadirah Damseh, Danita Velasco, Irene Valenzuela, Marta Codina-Solà, Catherine Ziats, Jaclyn Have, Katie Clarkson, Dora Steel, Manju Kurian, Katy Barwick, Diana Carrasco, Aditi I. Dagli, M. J. M. Nowaczyk, Miroslava Hančárová, Šárka Bendová, Darina Prchalova, Zdeněk Sedláček, Alica Baxová, Catherine Bearce Nowak, Jessica Douglas, Wendy K. Chung, Nicola Longo, Konrad Platzer, Chiara Klöckner, Luisa Averdunk, Dagmar Wieczorek, Ilona Krey, Christiane Zweier, Andre Reis, Tugce Balci, Marleen Simon, Hester Y. Kroes, Antje Wiesener, Georgia Vasileiou, Nikolaos M. Marinakis, Danai Veltra, Christalena Sofocleous, Konstantina Kosma, Joanne Traeger Synodinos, Konstantinos A. Voudris, Marie-Laure Vuillaume, Paul Gueguen, Nicolas Derive, Estelle Colin, Clarisse Battault, Billie Au, Martin Delatycki, Mathew Wallis, Lyndon Gallacher, Fatma Majdoub, Noor Smal, Sarah Weckhuysen, An-Sofie Schoonjans, R. Frank Kooy, Marije Meuwissen, Benjamin T. Cocanougher, Kathryn Taylor, Carolyn E. Pizoli, Marie T. McDonald, Philip James, Elizabeth R. Roeder, Rebecca Littlejohn, Nicholas A. Borja, Willa Thorson, Kristine King, Radka Stoeva, Manon Suerink, Esther Nibbeling, Stephanie Baskin, Gwenaël L. E. Guyader, Julie Kaplan, Candace Muss, Deanna Alexis Carere, Elizabeth J. K. Bhoj, Laura M. Bryant

**Affiliations:** 1 Department of Human Genetics, Children’s Hospital of Philadelphia, Philadelphia, PA USA; 2 Perelman School of Medicine, University of Pennsylvania, Philadelphia, PA USA; 3 Service de Génétique, CHU de Tours, Tours, France; 4 UMR1253, iBrain, Inserm, University of Tours, Tours, France; 5 Division of Clinical and Metabolic Genetics, Hospital for Sick Children, University of Toronto, Toronto, ON Canada; 6 Children’s Nebraska, University of Nebraska Medical Center, Omaha, NE USA; 7 Department of Clinical and Molecular Genetics and Rare Disease Unit Hospital Vall d’Hebron, Barcelona, Spain; 8 Medicine Genetics Group, Vall Hebron Research Institute, Barcelona, Spain; 9 Shodair Children’s Hospital, Helena, MT USA; 10 Greenwood Genetic Center, Greenwood, SC USA; 11 UCL Great Ormond Street Institute of Child Health, London, UK; 12 Department of Clinical Genetics, Cook Children’s Hospital, Fort Worth, TX USA; 13 Orlando Health, Arnold Palmer Hospital For Children, Orlando, FL USA; 14 McMaster University Medical Centre, Hamilton, ON Canada; 15 Charles University Second Faculty of Medicine and University Hospital Motol, Prague, Czech Republic; 16 Charles University First Faculty of Medicine and General University Hospital, Prague, Czech Republic; 17 Division of Genetics and Metabolism, Massachusetts General Hospital for Children, Boston, MA USA; 18 Harvard Medical School, Boston, MA USA; 19 Boston Children’s Hospital, Boston, MA USA; 20 University of Utah, Salt Lake City, UT USA; 21 Institute of Human Genetics, University of Leipzig Medical Center, Leipzig, Germany; 22 Institute of Human Genetics, Heinrich-Heine-University Düsseldorf, Medical Faculty, Düsseldorf, Germany; 23 Institute of Human Genetics, Universitätsklinikum Erlangen, Friedrich-Alexander-Universität Erlangen-Nürnberg (FAU), 91054 Erlangen, Germany; 24 Department of Human Genetics, Inselspital Bern, University of Bern, Bern, Switzerland; 25 University of Western Ontario, London, ON Canada; 26 Department of Genetics, University Medical Center, Utrecht, Netherlands; 27 Laboratory of Medical Genetics, St. Sophia’s Children’s Hospital, National and Kapodistrian University of Athens, Athens, Greece; 28 Second Department of Paediatrics, University of Athens, ‘P & A Kyriakou’ Children’s Hospital, Athens, Greece; 29 Laboratoire de Biologie Médicale Multi-Sites SeqOIA, Paris, France; 30 Service de Génétique Médicale, CHU d’Angers, Angers, France; 31 University of Calgary, Calgary, AB Canada; 32 Victorian Clinical Genetics Services, Murdoch Children’s Research Institute, Parkville, VIC Australia; 33 Department of Paediatrics, University of Melbourne, Melbourne, VIC Australia; 34 Tasmanian Clinical Genetics Service, Tasmanian Health Service, Hobart, TAS Australia; 35 School of Medicine and Menzies Institute for Medical Research, University of Tasmania, Hobart, TAS Australia; 36 Applied and Translational Neurogenomics Group, VIB Center for Molecular Neurology, Antwerp, Belgium; 37 Applied and Translational Neurogenomics Group, Department of Biomedical Sciences, University of Antwerp, Antwerp, Belgium; 38 Medical Genetics Department, University Hedi Chaker Hospital of Sfax, Sfax, Tunisia; 39 Department of Pediatric Neurology, University Hospital Antwerp, Antwerp, Belgium; 40 Translational Neurosciences, Faculty of Medicine and Health Science, University of Antwerp, Antwerp, Belgium; 41 NEURO Research Centre of Excellence, University of Antwerp, Antwerp, Belgium; 42 Department of Pediatrics, Duke University Hospital, Durham, NC USA; 43 Center of Medical Genetics, Antwerp University Hospital/University of Antwerp, Edegem, Belgium; 44 Division of Pediatric Neurology, Duke University Hospital, Durham, NC USA; 45 Division of Medical Genetics, Duke University Hospital, Durham, NC USA; 46 DMG Children’s Rehabilitative Services, Phoenix, AZ USA; 47 Department of Pediatrics, Baylor College of Medicine, San Antonio, TX USA; 48 John T. Macdonald Foundation Department of Human Genetics, University of Miami Miller School of Medicine, Miami, FL USA; 49 Genetics Department, Mary Bridge Children’s Hospital, Multicare Health System, Tacoma, WA USA; 50 Medical genetics department, Centre Hospitalier, Le Mans, France; 51 Department of Clinical Genetics, Leiden University Medical Center (LUMC), Leiden, The Netherlands; 52 Department of Molecular and Human Genetics, Baylor College of Medicine, Houston, TX USA; 53 Service de Génétique médicale, Centre Labellisé Anomalies du Développement-Ouest Site, Poitiers, France; 54 Nemours Children’s Health, Wilmington, DE USA; 55 GeneDx, Gaithersburg, MD USA; 56 Steve and Cindy Rasmussen Institute for Genomic Medicine, Nationwide Children’s Hospital, Columbus, OH, USA

**Keywords:** Neurodevelopmental disorders, Medical genetics

## Abstract

Bryant-Li-Bhoj syndrome (BLBS), which became OMIM-classified in 2022 (OMIM: 619720, 619721), is caused by germline variants in the two genes that encode histone H3.3 (*H3-3A*/*H3F3A* and *H3-3B*/*H3F3B*) [1–4]. This syndrome is characterized by developmental delay/intellectual disability, craniofacial anomalies, hyper/hypotonia, and abnormal neuroimaging [1, 5]. BLBS was initially categorized as a progressive neurodegenerative syndrome caused by de novo heterozygous variants in either *H3-3A* or *H3-3B* [1–4]. Here, we analyze the data of the 58 previously published individuals along 38 unpublished, unrelated individuals. In this larger cohort of 96 people, we identify causative missense, synonymous, and stop-loss variants. We also expand upon the phenotypic characterization by elaborating on the neurodevelopmental component of BLBS. Notably, phenotypic heterogeneity was present even amongst individuals harboring the same variant. To explore the complex phenotypic variation in this expanded cohort, the relationships between syndromic phenotypes with three variables of interest were interrogated: sex, gene containing the causative variant, and variant location in the H3.3 protein. While specific genotype-phenotype correlations have not been conclusively delineated, the results presented here suggest that the location of the variants within the H3.3 protein and the affected gene (*H3-3A* or *H3-3B)* contribute more to the severity of distinct phenotypes than sex. Since these variables do not account for all BLBS phenotypic variability, these findings suggest that additional factors may play a role in modifying the phenotypes of affected individuals. Histones are poised at the interface of genetics and epigenetics, highlighting the potential role for gene-environment interactions and the importance of future research.

## Introduction

Bryant-Li-Bhoj syndrome (BLBS) (OMIM: 619720, 619721) is a multi-system disorder with profound neurodevelopmental and neurodegenerative phenotypes [1–4]. Germline variants in either *H3-3A*/*H3F3A* or *H3-3B*/*H3F3B* cause BLBS. Both *H3-3A* and *H3-3B* are highly intolerant to missense variants, with Genome Aggregation Database (gnomAD) v2.1.1 missense constraint metric z-scores of 3.16 and 2.88, respectively, where a z-score >2 indicates that a gene is highly intolerant to missense variants. gnomAD v2.1.1 is the most recent release with constraint metrics for these two genes [5]^.^ Additionally, only one variant (M120K) observed in affected individuals is present in the non-neurologic phenotype gnomAD v2.1.1 release (Supplementary Fig. 1), which may be a technical mapping error, as it is only present on one strand and did not meet the previous Exome Aggregation Consortium (ExAC) reporting criteria [1]. This gnomAD analysis supports that reported BLBS variants are causative, rather than expected variation within the population.

In all prior reports, BLBS is reported to affect both male and females equally. Notably, *H3-3A* and *H3-3B* are located on autosomes 1 and 17, respectively. Even in cases in which a gene implicated in Mendelian neurodevelopmental disorders (NDDs) is on an autosome, there exists a 2–4:1 NDD diagnostic discrepancy rate between males and females in the United States [6, 7], supporting the exploration of sex as a contributor to phenotypic heterogeneity in BLBS.

**Fig. 1:**
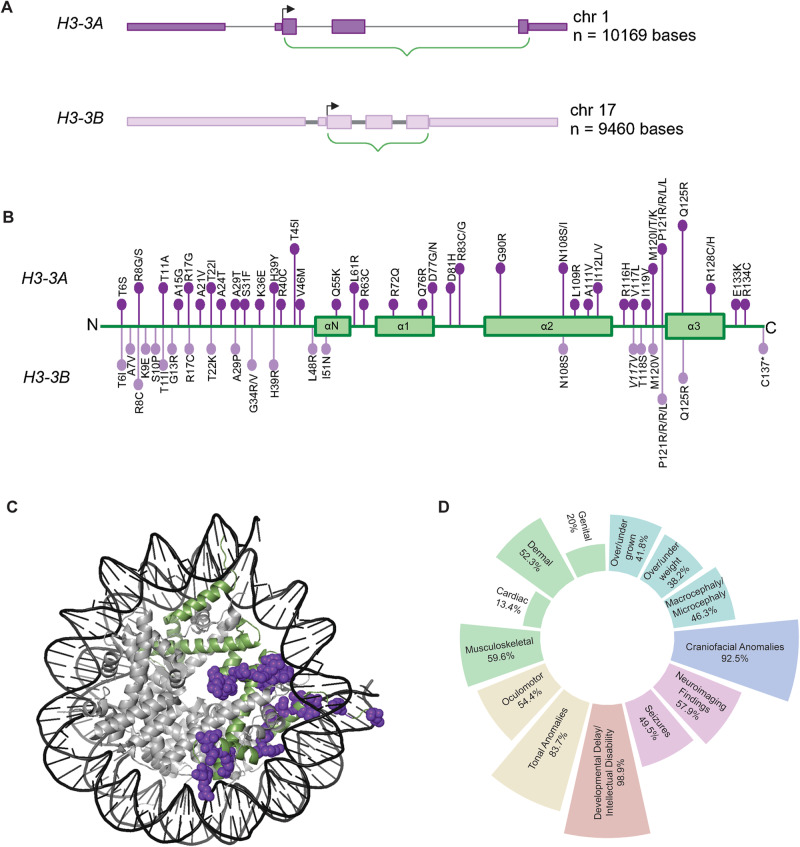
BLBS genotype and phenotype overview. **A** 2D diagram of the genes that encode the histone H3.3 protein – *H3-3A* (top - ENST00000366815) and *H3-3B* (bottom – ENST00000254810). Green brackets and thicker boxes represent the coding sequence. Arrows represent the transcription start sites. **B** 2D diagram of histone H3.3 protein (green), including the location of the four alpha helices. Lollipops show the *H3-3A* derived (top) and the *H3-3B* derived (bottom) heterozygous germline variants. Length of lollipop corresponds to number of individuals who harbor a variant at that residue (e.g. *H3-3A* p.T45I represents four individuals with BLBS and H3-3A p.V46M represents one individual with BLBS). **C** 3D in silico structural model of the H3.3-containing nucleosome (PDB: 5X7X) with the two copies of H3.3 in green; other histones in gray; and DNA in black. The location of heterozygous germline variants in the crystallized histone core are highlighted in purple. **D** Circular boxplot visualizing BLBS phenotypes. Cyan = growth (height, weight and head circumference); blue = craniofacial anomalies; pink = abnormal neuroimaging findings and seizures; red = developmental milestones; yellow = tone anomalies and oculomotor features; green = review of systems.

From a fundamental biology perspective, it is important to consider which of the two H3.3 encoding genes (*H3-3A* or *H3-3B)* is perturbed, given unique properties that distinguish them from most other protein-coding genes. Systemic knockout of each gene individually in murine models leads to distinct phenotypes, suggesting that these genes are not functionally redundant [8–11]. Further, while *H3-3A* is constitutively expressed, *H3-3B* is expressed in response to cellular stress and stimuli. This differential expression impacts H3.3 incorporation into the nucleosome. Further, these genes contain distinct exonic and intronic sequences, yet encode an identical H3.3 protein (Fig. 1A). This absolute conservation at the protein level, in spite of the two independent genes and four alleles, is a rare protein phenomenon, but common amongst histones [12]. This underlying histone biology suggests that each gene has distinct functional significance and that phenotypic variation observed across individuals with BLBS might be due in part to whether *H3-3A* or *H3-3B* is affected.

The H3.3 protein encoded by these two genes is comprised of a disordered tail, four α helices, and two loop domains. As in prior reports, the disordered tail is defined as amino acids (AA) 1–43 and the core (four α helices and two loop domains) as AA 44–135 (Fig. 1B), with the initiating methionine excluded from numbering [1, 4, 13–18]. Phenotypic variability could also be affected by which protein domain, specifically the tail or core, is affected by the variant. Certain “hotspot” somatic variants in H3.3, which are restricted to residues that include H3.3 p.K27M and p.G34R/V, cause pediatric brain tumors [17–22]. While these somatic variants are restricted to the H3.3 tail, the causative germline variants in BLBS are distributed throughout H3.3 (Fig. 1B). Currently, there is no evidence that germline H3.3 variants are oncogenic, but this is an area of ongoing investigation [4, 23].

It also important to distinguish between the histone core and tail because of the histone code. The histone code, written by post-translational modifications (PTMs) of amino acid residues that play a fundamental role in transcriptional regulation, is most commonly associated with the modification of residues on the N-terminal tail of H3.3 [24]. It is unsurprising that germline perturbation of residues in the tail are associated with disease pathogenesis [1]. However, affected individuals demonstrate that germline variants in the core also cause BLBS. Interestingly, in Tessadori-Bicknell-van Haaften (TBvH) NDD, caused by germline variants in histone H4, all known variants are restricted to the histone core [25, 26]. The mechanisms by which germline variants in the H3.3 and H4 cores cause BLBS and TBvH NDD remain poorly understood.

Here, 38 previously unpublished, unrelated individuals with heterozygous germline variants in *H3-3A*/*H3F3A* or *H3-3B*/*H3F3B* nearly double the number of known individuals affected by BLBS. All 58 previously published individuals are included in this analysis, totaling 96 individuals, which enables the interrogation of the effect of 1) the sex of the individual; 2) the gene harboring the germline variant; or 3) the location of the variant in the histone tail versus the histone core on BLBS phenotypes [1–4]. Then, potential genotype-phenotype relationships are interrogated by sub-stratifying the cohort into 1) individuals with the same missense variant in different genes; 2) individuals with different missense variants affecting the same residue in different genes; and 3) individuals with either germline or somatic variants in different genes affecting the same residue. As with many ultra-rare diseases, there are analytical limits that arise from the small number of affected individuals. Since a single individual can drastically affect statistical significance, it is cautioned that utilizing p-values to denote significance may not reflect the trends within the overall population [27, 28], statistical significance may not always reflect biological significance. For these reasons, trends, rather than statistical significance, are reported here.

## Subjects and methods

The Institutional Review Board of the Children’s Hospital of Philadelphia approved this study. Informed consent was obtained from all individuals included in the study. Individuals were referred by clinicians to EJKB through GeneMatcher [29]. Individual phenotypes and genetic sequencing information were provided by the referring clinicians. Analyses and graphs were made in Microsoft Excel, GraphPad Prism v8, and R v4.3.2 using circular barplot code from R-graph-gallery (https://github.com/holtzy/R-graph-gallery). Graphics were generated with BioRender.

### Phenotyping criteria

The denominators presented in Table 1 for each sub-analysis are not always equal to the total number of individuals with BLBS (96). A denominator less than 96 reflects the subset of individuals for whom phenotypic information for a particular sub-analysis was available or consideration of developmental stage. For instance, when analyzing attainment of independent sitting, walking or first words, individuals who have not yet surpassed the expected window of achievement for these milestones are excluded (e.g. an individual who is 18 months old would be included in the independent sitting metric, but not the independent walking or speech metrics).

**Table 1:** BLBS clinical phenotypes.

Demographics	
Age at evaluation	2 months – 39 years
**Sex**	Males – 47
	Females – 49
**Growth**	
Height (>95th percentile)	6/91 (7%)
Height (≤5th percentile)	32/91 (35%)
Weight (≥95th percentile)	14/76 (18%)
Weight (≤5th percentile)	15/76 (20%)
Macrocephaly (≥95th percentile)	14/95 (15%)
Microcephaly (≤5th percentile)	30/95 (32%)
Craniofacial anomalies	86/93 (92%)
Neuroimaging findings	44/76 (58%)
Corpus collosum malformation/dysgenesis	28/76 (37%)
Dilated ventricles	6/76 (8%)
Asymmetry	4/76 (5%)
**Neurodevelopment**	
Developmental delay/intellectual disability	94/95 (99%)
Seizures	45/91 (49%)
Delayed/No sitting (>12 months)	33/65 (51%)
Delayed/No walking (>20 months)	59/75 (79%)
Speaks at least one word (>20 months)	50/84 (60%)
**Muscle tone anomalies**	
Hypotonia	57/92 (62%)
Hypertonia	11/92 (12%)
Axial hypotonia, peripheral hypertonia	9/92 (10%)
Oculomotor	49/90 (54%)
Strabismus	32/90 (36%)
Astigmatism	7/88 (8%)
**Review of systems**	
Musculoskeletal	56/94 (60%)
Scoliosis	20/94 (21%)
Lordosis/Kyphosis	4/94 (4%)
Ligamentous laxity	21/94 (22%)
Cardiac	11/82 (13%)
Dermal	46/88 (52%)
Eczema	6/88 (7%)
Nipple anomalies	15/88 (17%)
Fetal finger pad	14/88 (16%)
Genital	17/85 (20%)

In alignment with field standards, overgrowth (height/weight) and macrocephaly were defined as measurements that were equal to or above 95^th^ percentile, or greater than 2 standard deviations (SD) above the mean, compared to age- and sex-matched controls [30]. Undergrowth (height/weight) and microcephaly were similarly defined as measurements that were equal to or below the 5^th^ percentile, or greater than 2 SD below the mean, compared to age- and sex-matched controls.

Delayed attainment of developmental milestones was defined based on established developmental trajectories within pediatrics [31]. An individual was classified as demonstrating delayed independent sitting if they had not yet achieved that milestone at 12 months of age. Delayed independent walking was identified if an individual had not yet achieved that milestone at 20 months of age. Delayed speech was indicated if an individual had not yet achieved their first word at 20 months of age.

### PyMOL in silico 3D structural protein modeling

Utilizing PyMOL Molecular Graphics System Version 2.5.5, the crystallized structure of the nucleosome containing H3.3 at 2.18 Å resolution was imported from the Research Collaboratory for Structural Bioinformatics Protein Data Bank by referencing ID 5X7X [32]. The H3.3 protein sequence identity was verified by cross-referencing UniProt Knowledgebase sequences (Human H3.3 - P84243). H3.3 is color-coded “smudge green” and denoted as green; all other histones are color-coded “gray70” and denoted as gray; the DNA double helix is color-coded “gray10” and denoted as black; and BLBS-causing variants are color-coded “purpleblue” and denoted as purple.

## Results

Thirty-eight previously unpublished, unrelated individuals with BLBS, along with the 58 previously reported individuals, represent a global cohort of 96 individuals who harbor 70 unique causative variants (Fig. 1B and C, Table 1, Supplementary Table 1) [1–4]. Individuals range in age from 10 weeks to 39 years at the time of their most recent evaluation, and include 47 males and 49 females (Table 1, Supplementary Table 1). In total, 65 individuals harbor variants in *H3-3A* and 31 individuals harbor variants in *H3-3B*. At the time of this report, all *H3-*3*A* variants are heterozygous de novo missense variants when parents are available, though inheritance was undetermined for three individuals (Supplementary Table 1). Conversely, while most variants in *H3-3B* are heterozygous de novo missense variants, more variability in variant type and modes of inheritance was observed. One individual harbors a variant that is synonymous in the canonical *H3-3B* transcript and leads to a stop-gain in a non-canonical transcript *(H3-3B* p.V117V/S147*) [1]. Another individual harbors a two-nucleotide deletion that ablates the stop codon *(H3-3B* p.C136*ext9) (Fig. 1B, Supplementary Table 1) [3]. Two individuals with variants in *H3-3B* have unknown inheritance and one individual has a maternally inherited *H3-3B* heterozygous missense variant (*H3-3B* p.N108S) (Supplementary Table 1). The referring geneticist has confirmed the maternal genotype and is working to fully phenotype and genotype this individual’s siblings, mother, and maternal grandparents.

## BLBS phenotypic variability

BLBS is predominantly characterized by developmental delay/intellectual disability (DD/ID), growth anomalies, craniofacial anomalies, abnormal neuroimaging, and hypo/hypertonia (Fig. 1D, Table 1, Supplementary Table 1). Phenotypic heterogeneity is noted across the BLBS population. For example, individuals may present with microcephaly, macrocephaly, or a head circumference within the normal range (Table 1, Supplementary Table 1). Potential sources underlying this variation were interrogated by stratifying by 1) the sex of the individual; 2) which of the two genes harbors the variant; and 3) the location of the variant in the histone tail or core.

### BLBS and growth

**Fig. 2:**
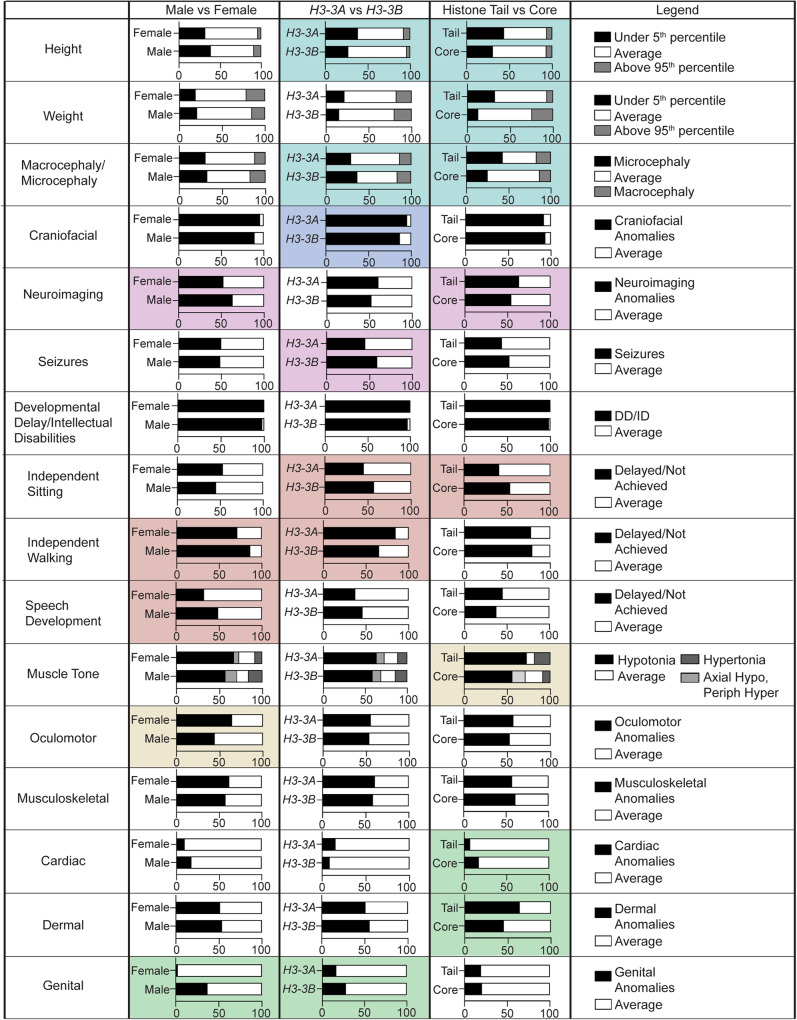
Interrogating the relationship between BLBS phenotypes and sex, gene, and variant location in H3.3. Phenotypic categories (rows) analyzed across all 96 individuals with BLBS include growth; craniofacial features; neuroimaging findings and seizures; attainment of developmental milestones; tone anomalies; and general review of systems. Phenotypic analyses were performed by stratifying the cohort of individuals (columns) based on their sex (reported as male or female) (column 1); on the localization of their causative variant to either *H3-3A* or *H3-3B* (column 2); or on the location of their causative variant to the histone tail or histone core (column 3). Each graph is representative of the percentages of individuals with BLBS for which this category was reported. The colored cells highlight stratifications emphasized in the text. The color-coding is based on the phenotypic overview in Fig. 1D, where cyan = growth (height, weight and head circumference); blue = dysmorphic craniofacial features; pink = neuroimaging findings and seizures; red = developmental milestones; yellow = tone anomalies and oculomotor features; green = review of systems.

Over half of individuals with BLBS demonstrate typical age- and sex-based growth (Table 1, Fig. 1D, Supplementary Table 1). Forty-one percent of individuals have height trajectories outside of the normal range. Most of these individuals exhibit undergrowth (35%) (Table 1, Supplementary Table 1). This trend holds when the population is stratified by sex or gene (Fig. 2, Supplementary Table 1). When variants are stratified by location in H3.3, this trend is even more pronounced, with 44% of individuals harboring variants in the tail presenting with undergrowth while only 6% presenting with overgrowth (Fig. 2, Supplementary Table 1). Even though individuals with variants in the core demonstrate a similar distribution between overgrowth and undergrowth, 63% of individuals with variants in the core are reported to be of average height, compared to 50% of those with variants in the tail (Fig. 2, Supplementary Table 1). Similarly, individuals with variants in *H3-3A* (46%) are more likely to present with undergrowth or overgrowth than those with variants in *H3-3B* (31%).

Of the 38% of individuals with weight metrics reported outside of the normal range, 14% of reported weights were ≤5^th^ and 14% of reported weights were ≥95^th^ percentile (Table 1, Supplementary Table 1). As with height, individuals with variants in the histone tail show a similar phenotypic distribution in relation to their weight, with more individuals being underweight (32%) than overweight (7%) (Fig. 2, Supplementary Table 1). Interestingly, the pattern is inverted for individuals with variants in the core: 25% of individuals are reported as being overweight while 12% are reported as being underweight (Fig. 2, Supplementary Table 1).

### BLBS and craniofacial development

In addition to growth trajectories outside of the standard range for age and sex, 92% of individuals with BLBS present with craniofacial anomalies (Table 1, Fig. 1D, Supplementary Table 1). Commonly reported features are broad/prominent forehead, broad nasal bridge, thin upper lip, hypertelorism, epicanthal folds, and ear and tooth anomalies.Dysmorphic features are slightly more common in individuals with variants in *H3-3A* (95%) than *H3-3B* (86%) (Fig. 2, Supplementary Table 1), though it is important to note that these individuals have all been phenotyped by different geneticists and the assessment of facial feature analysis is strongly influenced by the ancestral and ethnic background of both the evaluator and the affected individual [33].

Notably, 46% of individuals with craniofacial anomalies present with concurrent microcephaly or macrocephaly (Table 1, Fig. 1D, Supplementary Table 1). Among these individuals, microcephaly is more common than macrocephaly (32 and 15%, respectively), which is consistent across all variables interrogated (Table 1). The occurrence of micro- or macrocephaly is most different between variants in the tail or core (60 and 38%, respectively) (Fig. 2). Microcephaly is more common in individuals with variants in the tail (43%) than the core (25%). Discrepancies in the prevalence of micro- and macrocephaly also depend upon the affected gene (43% *H3-3A* and 53% *H3-3B*, respectively) (Fig. 2).

### BLBS and neuroradiological imaging

Seventy-nine percent of individuals with BLBS underwent diagnostic magnetic resonance imaging (MRI). Fifty-eight percent of individuals with a reported MRI were diagnosed with at least one abnormal finding (Table 1). While not all referring clinicians elaborated on the MRI results, common findings included delayed myelination or hypomyelination; dysgenesis of the corpus collosum; dilated ventricles; and hemispheric asymmetry across multiple structures (Table 1, Supplementary Table 1). These findings were expanded upon by Alves et al., where 18 MRIs from previously reported individuals with BLBS were carefully analyzed [1, 34]. Within this subgroup, 72% of individuals presented with small posterior fossa, 28% presented with dysgenesis of the corpus collosum, and 44% presented with cortical developmental abnormalities [34].

When analyzing MRI findings beyond these 18 individuals, abnormal findings were more commonly reported for individuals with variants in the tail (63%) than for individuals with variants in the core (54%). Abnormal findings were seen in more males (63%) than females (53%). Forty nine percent of individuals also present with seizures (Table 1). Of these individuals, 20% experienced febrile seizures. Seizures were more frequently reported in individuals harboring variants in *H3-3B* (59%) than those harboring variants in *H3-3A* (45%) (Fig. 2).

### BLBS and developmental milestones

Ninety-nine percent of individuals with BLBS have DD/ID (Table 1, Fig. 1D, Supplementary Table 1). The only individual not reported to have DD/ID harbors the *H3-3B* p.V117V/S147* variant [1]. While he is the only individual reported with a synonymous variant, he exhibited delayed attainment of speech (first word at 24 months); presented with an “expressive language disorder with neurologic progression” when evaluated at 15 years; and had neuroimaging anomalies consistent with the other individuals in this cohort.

In addition to DD/ID, many individuals have co-existing neurodevelopmental diagnoses, including 8% with autism spectrum disorder, 3% with attention deficit disorder, and 3% with anxiety diagnoses. Others have neurobehavioral diagnoses, including behavioral issues (4%) and stereotyped repetitive movements (7%) (Supplementary Table 1). None of these phenotypes are correlated to sex, gene, or location (Fig. 2, Supplementary Table 1).

A subset of individuals displayed delays in developmental milestones including independent sitting, independent walking, and/or speech development (Table 1, Fig. 1D, Supplementary Table 1). Individuals with variants in the tail (59%) more frequently had delayed or yet to be achieved independent sitting compared to individuals with variants in the core (47%) (Fig. 2). Individuals with variants in *H3-3A* (56%) more frequently had delayed or yet to be achieved independent sitting compared to individuals with variants in *H3-3B* (43%) (Fig. 2). More individuals harboring variants in *H3-3A* (85%) had delayed or yet to be achieved independent walking when compared to individuals harboring variants in *H3-3B* (65%). Differences were not seen between variants in the tail versus core (Fig. 2). Sex captured some phenotypic variability related to independent walking (86% of males had delayed or yet to be achieved independent walking compared to 71% females) and speech development (33% of females had not yet achieved one word compared to 49% of males) (Fig. 2).

### BLBS and hyper/hypotonia

Eighty-four percent of individuals present with hypotonia, hypertonia, or a combination of axial hypotonia with peripheral hypertonia (Table 1, Fig. 1D, Supplementary Table 1). Sixty-two percent of individuals presented with hypotonia whereas 12% presented with hypertonia (Table 1, Fig. 2). Five of the reported 55 individuals presented with resolved hypotonia at their most recent evaluation. Interestingly, 10% of individuals have concordant axial hypotonia and peripheral hypertonia, or dystonia, which is exclusively present in individuals with variants in the core (Fig. 2, light gray). Hypotonia is also more commonly reported for individuals with variants in the tail (73%) compared to those with variants in the core (56%) (Fig. 2). While not directly queried here, two individuals were reported to demonstrate an ataxic gait, while Okur et al. reported that individuals in their cohort universally presented with gait anomalies [1, 3].

In conjunction with global tone abnormalities, 54% of individuals report oculomotor dysfunction, 82% of whom demonstrate eye rolling and strabismus, which may be attributed to abnormal muscle tone (Table 1, Fig. 1D, Supplementary Table 1). More females (64%) present with oculomotor dysfunction than males (44%) (Fig. 2).

### BLBS and review of systems

Beyond the neurological features, individuals also variably present with phenotypes resulting in abnormalities within the musculoskeletal, dermatologic, cardiac, and genital systems (Fig. 1D). Over half of individuals (58%) present with musculoskeletal anomalies such as club foot, scoliosis, hip dysplasia, subluxation of various joints/hypermobility, kyphosis, and femoral anteversion (Figs. 1D and 2, Table 1, Supplementary Table 1).

Fifty-three percent of individuals present with dermal phenotypes such as eczema, hypoplastic nails, fetal finger pads, nipple abnormalities and 2/3 toe syndactyly (Fig. 1D, Table 1, Supplementary Table 1). Dermal features are more likely found in individuals with variants in the tail (64%) than the core (45%) (Fig. 2).

Fourteen percent of individuals present with cardiac anomalies, including atrial septal defects (Fig. 1D). Cardiac anomalies are twice as likely to be reported in individuals with core variants (17%) than tail variants (7%) (Table 1, Fig. 2, Supplementary Table 1).

Twenty percent of individuals present with genital anomalies (Fig. 1D, Table 1, Supplementary Table 1). Genital anomalies are more often reported in males (37%) than females (2%), though this may be related to the necessity of intervention associated with a particular anomaly, such as cryptorchidism. More individuals with variants in *H3-3B* (28%) present with genital anomalies compared to individuals with variants in *H3-3A* (17%).

While urinary anomalies were not specifically queried here, some clinicians reported phenotypes such as small right kidney, horseshoe kidney, solitary kidney, nephrocalcinosis, and chronic urinary tract infections (Supplementary Table 1). This suggests that genitourinary surveillance may be important for individuals with BLBS going forward.

## Exploration of potential genotype-phenotype correlations in BLBS

**Fig. 3:**
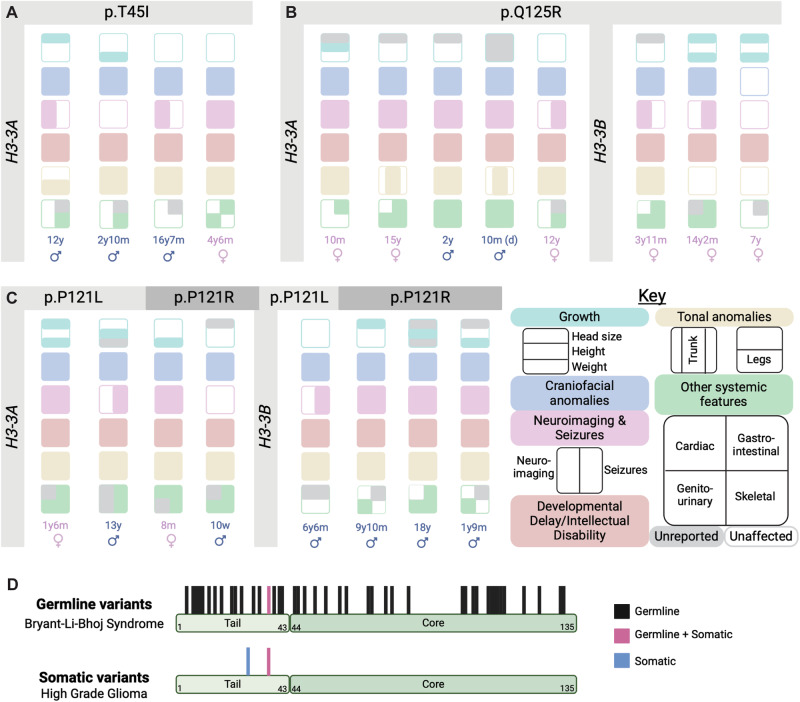
BLBS-associated phenotypic heterogeneity amongst individuals with similar genotypes. **A**–**C** Representation of the phenotypic variation across individuals who (**A**) share the same missense variant in the same residue of the same gene, (**B**) the same missense variant in the same residue of different genes, or (**C**) different missense variants affecting the same residue of different genes. The color-coding in the key corresponds to the phenotypic overview in Fig. 1D and Fig. 2. **A** Representation of the phenotypic variation across the four individuals who share the *H3-3A* p.T45I variant. **B** Representation of phenotypic variation across the eight individuals who share the H3.3 p.Q125R variant. Five individuals harbor a nucleotide substitution in *H3-3A* while three individuals harbor a nucleotide substitution in *H3-3B*. **C** Representation of phenotypic variation across the eight individuals who harbor variants affecting the H3.3 p.P121 residue. Four individuals harbor a nucleotide substitution in *H3-3A* (leading to either p.P121L or p.P121R missense variants) while four individuals harbor a nucleotide substitution in *H3-3B* (leading to either p.P121L or p.P121R missense variants). **D** Phenotypic variation across individuals with BLBS-causing germline variants throughout the disordered histone tail region and histone core (top) compared to hotspot high grade glioma-causing somatic mutations (bottom). Black = amino acids with associated germline variants; magenta = amino acids with associated germline and somatic variants; blue = amino acids with associated somatic variants.

Potential genotype-phenotype correlation was explored given our hypothesis that individuals harboring similar variants would present with similar phenotypes. A similar analysis was previously performed for the four individuals harboring *H3-3A* p.T45I variants, which showed phenotypic variation (Fig. 3A) [1]. With this cohort expansion, additional analyses were performed to explore the possibility of a genotype-phenotype correlation in other subgroups who harbored 1) the same variant in the same residue of different genes (*H3-3A* vs *H3-3B)* (Fig. 3B); 2) different variants in the same residue of different genes (Fig. 3C); and 3) variants arising in germline versus somatic cells (Fig. 3D).

### Same missense variant, same residue, different genes

Eight individuals harbor the H3.3 p.Q125R variant (Fig. 3B). This protein alteration results from a nucleotide change in *H3-3A* for 5 individuals and a change in *H3-3B* for 3 individuals. In this subcohort, individuals span the ages of 10 months to 15 years. All individuals present with DD/ID. Only two individuals exhibit overgrowth, a phenotype that seems to segregate with variants in *H3-3A* in this sub-cohort (Fig. 3B). Conversely, two individuals present with macrocephaly, a phenotype only associated with *H3-3B* in this sub-cohort (Fig. 3B). Finally, all five individuals with the *H3-3A* variant present with seizures, while seizures are only reported in one individual with an *H3-3B* variant (Fig. 3B).

### Different missense variant, same residue, different genes

Eight unrelated individuals between the ages of 2.5 months to 18 years harbor variants that affect the p.P121 residue (Fig. 3C). Four individuals harbor a c.365C>T change, resulting in p.P121R variants, while the other four individuals harbor a c.365C>G change, resulting in p.P121L variants. All individuals have DD/ID and either hypotonia or a combination of axial hypotonia and peripheral hypertonia (Fig. 3C). Additionally, seven of the eight individuals present with seizures (Fig. 3C, Supplementary Table 1).

### Different cells of origin

BLBS arises from germline variants in H3.3 while high grade gliomas arise from somatic variants in H3.3. These somatic variants have a specific genotype-phenotype relationship, exhibiting precise spatiotemporal relationships (Fig. 3D) [4, 22, 23]. H3.3 p.K27M somatic variants are associated with universally fatal diffuse midline gliomas in children under 12, while H3.3 p.G34R/V somatic variants are associated with diffuse hemispheric brain tumors, with age of diagnosis between 12–35 [4, 22, 23]. Two individuals with BLBS harbor germline variants affecting the p.G34 residue (p.G34R and p.G34V) [4]. Interestingly, these individuals harbor germline substitutions in *H3-3B*, while individuals with diffuse hemispheric brain tumors harbor somatic variants in *H3-3A* (Fig. 3D) [4]. Currently, there are no reported cases of individuals with BLBS with any oncologic diagnoses, including high grade gliomas.

## Discussion

With this expanded cohort of individuals, the genetic causes and resultant phenotypes of the BLBS population can be more thoroughly examined. These updated analyses highlight that the four most common features of BLBS are DD/ID, craniofacial anomalies, abnormal neuroimaging findings, and tonal anomalies (Fig. 1D, Table 1, Supplementary Table 1). However, the presentation of these features is highly variable across individuals, and the addition of each individual continues to deepen our understanding of the phenotypic spectrum of BLBS. The variability suggests that molecular testing will continue to play a role in diagnosing affected individuals, though a characteristic phenotype for individuals with BLBS may yet emerge.

The current stratification by sex, affected gene, or affected protein domain does not account for all phenotypic variation observed in individuals with BLBS. This suggests that the remainder of phenotypic heterogeneity may be attributed to other molecular mechanisms, such as the incorporation of H3.3 with the causative variant into nucleosomes, or the altered deposition of PTMs on H3.3, leading to a disrupted histone code and aberrant gene regulation [24, 35]. Additional functional work will be crucial both for diagnosis and the development of therapeutic interventions [36].

In ultra-rare Mendelian NDDs, every affected individual impacts the way translational research and medical communities understand a syndrome. Collaboration and data-sharing between groups around the world is imperative to ensure that the generous gift to medicine and science that each affected individual’s family makes shapes the trajectory of the field. In a five-year span from 2019 to 2024, we have moved from the first single-individual BLBS case report to now analyzing a cohort of almost 100 individuals, which has enabled the deep interrogation of trends. This patient-guided approach, coupled with ongoing functional work, will hopefully enable more conclusive guidance in the near-future.

### Follow-up phenotyping

This analysis of BLBS phenotypes highlights the need for repeat phenotyping of individuals throughout their lives. Longitudinal follow-up is currently only accessible for a few of the individuals presented in this cohort (*H3-3A* p.L61R, *H3-3B* p.P121R and p.Q125R) [1, 2, 3, 37]. In infancy, individuals who harbor the *H3-3B* p.P121R and p.Q125R variants were diagnosed with an unspecified overgrowth disorder but, at follow up years later, they presented with normal height or undergrowth [1, 37]. Longitudinal follow-up will also allow for direct comparison between evaluation timepoints, elucidating more detail about the temporal phenotype, including the previously established neurodegenerative component of this syndrome [1, 4]. This long-term follow-up could facilitate a deeper understanding of the tonal anomalies associated with BLBS. For some individuals, there is a progressive transition between generalized hypotonia to axial hypotonia with peripheral hypertonia, while for others, there is a trend of resolved hypotonia. This suggests that there may be an underlying neurological progression. Additionally, 25% of individuals are diagnosed with concurrent neurodevelopmental and neurobehavioral diagnoses. This concurrence can only be diagnosed once children reach a certain age or developmental stage. Identifying individuals with syndromic features in addition to neurobehavioral diagnoses would not only facilitate access to resources like behavioral intervention programs, but could also aid in the referral of these individuals for genetic testing [38]. This would be most beneficial in cases of individuals with less severe phenotypes who would not otherwise be referred for whole exome or genome sequencing.

Further, when individuals have access, and based on considerations such as the need for sedation, repeat neuroimaging could facilitate the management of progressive disease, as suggested in the 2023 Gene Reviews entry by Bryant and Bhoj [38]. Many individuals present with dysgenesis/hypoplastic/thinning corpus collosum as well as hypomyelination [34]. These phenotypes exhibit overlap with leukodystrophy disorders, which are characterized by structural brain and muscle tone anomalies [34, 39, 40]. Repeat brain MRIs could help determine whether this is a progressive neurodegenerative disorder, as is suggested by the tonal and the abnormal gait/progressive gait ataxia phenotype [3]. Further delineating the neurodegenerative characteristics of BLBS would help clinicians provide prognostic information to families.

Finally, somatic variants in H3.3 are driver mutations in cancers like high-grade gliomas [23]. Current phenotypic evaluations do not suggest that individuals with BLBS have a cancer predisposition. It is possible that some individuals have since received a cancer diagnosis, as malignancy was not a specific query of these surveys. Longitudinal phenotyping of individuals with BLBS could enable more definitive answers related to co-morbidities for individuals and their families.

### BLBS, not just de novo missense variants

In the initial characterization of BLBS, all individuals harbored heterozygous de novo missense variants. The individual with the reported synonymous *H3-3B* p.V117V variant was excluded from the phenotyping analysis at that time. It has since been confirmed that this individual harbors a synonymous variant in the canonical *H3-3B* transcript, which maps to a truncating variant in a non-canonical transcript [1]. Investigations into the implications of this variant on understanding the mechanism of pathogenesis in BLBS are ongoing. Additionally, an individual with a stop-loss variant in *H3-3B* was reported [3]. These variants highlight that the genotypic spectrum of the disorder is not isolated to missense variants.

This cohort also includes the first known individual with a BLBS-causing *inherited* variant. The individual harbors a maternally inherited p.N108S variant in *H3-3B* demonstrating that BLBS does not always arise from de novo variants. A full phenotypic evaluation of the individuals’ mother, maternal grandparents, and siblings is ongoing. Given the shared genetic background, families harboring inherited variants will play a crucial role in elucidating the additional genetic and environmental modifiers of BLBS.

In sum, this expanded cohort provides new detail about BLBS and extends its characterization to a neurodevelopmental and neurodegenerative disorder with variable multi-systemic effects. Ongoing functional work is needed to clearly determine how the factors reviewed here – sex, gene, and variant location – affect phenotypic variability. Additionally, future functional work is needed to elucidate how other factors impact the severity of this disorder. The information presented here, coupled with ongoing functional work, will aid in shortening the diagnostic odysseys for future individuals with BLBS and their families.

### Supplementary information

Supplementary Figure 1: Observed gnomAD v2.1.1 H3-3A and H3-3B variants overlaid with BLBS variants.

Supplementary Figure 1: Legend

Supplementary Table 1: BLBS Clinical Survey Responses

## Data Availability

All data analyzed in this study are available in the manuscript and represented in Supplementary Information.
