# Correction: Expanded phenotypic spectrum of neurodevelopmental and neurodegenerative disorder Bryant-Li-Bhoj syndrome with 38 additional individuals

**DOI:** 10.1038/s41431-024-01659-y

**Published:** 2024-07-26

**Authors:** Dana E. Layo-Carris, Emily E. Lubin, Annabel K. Sangree, Kelly J. Clark, Emily L. Durham, Elizabeth M. Gonzalez, Sarina Smith, Rajesh Angireddy, Xiao Min Wang, Erin Weiss, Annick Toutain, Roberto Mendoza-Londono, Lucie Dupuis, Nadirah Damseh, Danita Velasco, Irene Valenzuela, Marta Codina-Solà, Catherine Ziats, Jaclyn Have, Katie Clarkson, Dora Steel, Manju Kurian, Katy Barwick, Diana Carrasco, Aditi I. Dagli, M. J. M. Nowaczyk, Miroslava Hančárová, Šárka Bendová, Darina Prchalova, Zdeněk Sedláček, Alica Baxová, Catherine Bearce Nowak, Jessica Douglas, Wendy K. Chung, Nicola Longo, Konrad Platzer, Chiara Klöckner, Luisa Averdunk, Dagmar Wieczorek, Ilona Krey, Christiane Zweier, Andre Reis, Tugce Balci, Marleen Simon, Hester Y. Kroes, Antje Wiesener, Georgia Vasileiou, Nikolaos M. Marinakis, Danai Veltra, Christalena Sofocleous, Konstantina Kosma, Joanne Traeger Synodinos, Konstantinos A. Voudris, Marie-Laure Vuillaume, Paul Gueguen, Nicolas Derive, Estelle Colin, Clarisse Battault, Billie Au, Martin Delatycki, Mathew Wallis, Lyndon Gallacher, Fatma Majdoub, Noor Smal, Sarah Weckhuysen, An-Sofie Schoonjans, R. Frank Kooy, Marije Meuwissen, Benjamin T. Cocanougher, Kathryn Taylor, Carolyn E. Pizoli, Marie T. McDonald, Philip James, Elizabeth R. Roeder, Rebecca Littlejohn, Nicholas A. Borja, Willa Thorson, Kristine King, Radka Stoeva, Manon Suerink, Esther Nibbeling, Stephanie Baskin, Gwenaël L. E. Guyader, Julie Kaplan, Candace Muss, Deanna Alexis Carere, Elizabeth J. K. Bhoj, Laura M. Bryant

**Affiliations:** 1 Department of Human Genetics, Children’s Hospital of Philadelphia, Philadelphia, PA USA; 2 Perelman School of Medicine, University of Pennsylvania, Philadelphia, PA USA; 3 Service de Génétique, CHU de Tours, Tours, France; 4 UMR1253, iBrain, Inserm, University of Tours, Tours, France; 5 Division of Clinical and Metabolic Genetics, Hospital for Sick Children, University of Toronto, Toronto, ON Canada; 6 Children’s Nebraska, University of Nebraska Medical Center, Omaha, NE USA; 7 Department of Clinical and Molecular Genetics and Rare Disease Unit Hospital Vall d’Hebron, Barcelona, Spain; 8 Medicine Genetics Group, Vall Hebron Research Institute, Barcelona, Spain; 9 Shodair Children’s Hospital, Helena, MT USA; 10 Greenwood Genetic Center, Greenwood, SC USA; 11 UCL Great Ormond Street Institute of Child Health, London, UK; 12 Department of Clinical Genetics, Cook Children’s Hospital, Fort Worth, TX USA; 13 Orlando Health, Arnold Palmer Hospital For Children, Orlando, FL USA; 14 McMaster University Medical Centre, Hamilton, ON Canada; 15 Charles University Second Faculty of Medicine and University Hospital Motol, Prague, Czech Republic; 16 Charles University First Faculty of Medicine and General University Hospital, Prague, Czech Republic; 17 Division of Genetics and Metabolism, Massachusetts General Hospital for Children, Boston, MA USA; 18 Harvard Medical School, Boston, MA USA; 19 Boston Children’s Hospital, Boston, MA USA; 20 University of Utah, Salt Lake City, UT USA; 21 Institute of Human Genetics, University of Leipzig Medical Center, Leipzig, Germany; 22 Institute of Human Genetics, Heinrich-Heine-University Düsseldorf, Medical Faculty, Düsseldorf, Germany; 23 Institute of Human Genetics, Universitätsklinikum Erlangen, Friedrich-Alexander-Universität Erlangen-Nürnberg (FAU), 91054 Erlangen, Germany; 24 Department of Human Genetics, Inselspital Bern, University of Bern, Bern, Switzerland; 25 University of Western Ontario, London, ON Canada; 26 Department of Genetics, University Medical Center, Utrecht, Netherlands; 27 Laboratory of Medical Genetics, St. Sophia’s Children’s Hospital, National and Kapodistrian University of Athens, Athens, Greece; 28 Second Department of Paediatrics, University of Athens, ‘P & A Kyriakou’ Children’s Hospital, Athens, Greece; 29 Laboratoire de Biologie Médicale Multi-Sites SeqOIA, Paris, France; 30 Service de Génétique Médicale, CHU d’Angers, Angers, France; 31 University of Calgary, Calgary, AB Canada; 32 Victorian Clinical Genetics Services, Murdoch Children’s Research Institute, Parkville, VIC Australia; 33 Department of Paediatrics, University of Melbourne, Melbourne, VIC Australia; 34 Tasmanian Clinical Genetics Service, Tasmanian Health Service, Hobart, TAS Australia; 35 School of Medicine and Menzies Institute for Medical Research, University of Tasmania, Hobart, TAS Australia; 36 Applied and Translational Neurogenomics Group, VIB Center for Molecular Neurology, Antwerp, Belgium; 37 Applied and Translational Neurogenomics Group, Department of Biomedical Sciences, University of Antwerp, Antwerp, Belgium; 38 Medical Genetics Department, University Hedi Chaker Hospital of Sfax, Sfax, Tunisia; 39 Department of Pediatric Neurology, University Hospital Antwerp, Antwerp, Belgium; 40 Translational Neurosciences, Faculty of Medicine and Health Science, University of Antwerp, Antwerp, Belgium; 41 NEURO Research Centre of Excellence, University of Antwerp, Antwerp, Belgium; 42 Department of Pediatrics, Duke University Hospital, Durham, NC USA; 43 Center of Medical Genetics, Antwerp University Hospital/University of Antwerp, Edegem, Belgium; 44 Division of Pediatric Neurology, Duke University Hospital, Durham, NC USA; 45 Division of Medical Genetics, Duke University Hospital, Durham, NC USA; 46 DMG Children’s Rehabilitative Services, Phoenix, AZ USA; 47 Department of Pediatrics, Baylor College of Medicine, San Antonio, TX USA; 48 John T. Macdonald Foundation Department of Human Genetics, University of Miami Miller School of Medicine, Miami, FL USA; 49 Genetics Department, Mary Bridge Children’s Hospital, Multicare Health System, Tacoma, WA USA; 50 Medical genetics department, Centre Hospitalier, Le Mans, France; 51 Department of Clinical Genetics, Leiden University Medical Center (LUMC), Leiden, The Netherlands; 52 Department of Molecular and Human Genetics, Baylor College of Medicine, Houston, TX USA; 53 Service de Génétique médicale, Centre Labellisé Anomalies du Développement-Ouest Site, Poitiers, France; 54 Nemours Children’s Health, Wilmington, DE USA; 55 GeneDx, Gaithersburg, MD USA; 56 Steve and Cindy Rasmussen Institute for Genomic Medicine, Nationwide Children’s Hospital, Columbus, OH USA

**Keywords:** Neurodevelopmental disorders, Medical genetics

Correction to: *European Journal of Human Genetics* 10.1038/s41431-024-01610-1, published online 27 April 2024

An author was not named.

The missing author is: “Annick Toutain”

Her affiliation is:

27 Service de Génétique, CHU de Tours, Tours, France.

28 UMR1253, iBrain, Inserm, University of Tours, Tours, France.

