# Supplementary figures and images for: Expanded phenotypic spectrum of neurodevelopmental and neurodegenerative disorder Bryant-Li-Bhoj syndrome with 38 additional individuals
Source: Eur J Hum Genet. 2024 Apr 27;32(8):928–37. doi: 10.1038/s41431-024-01610-1 (PMC11291762; doi:10.1038/s41431-024-01610-1)

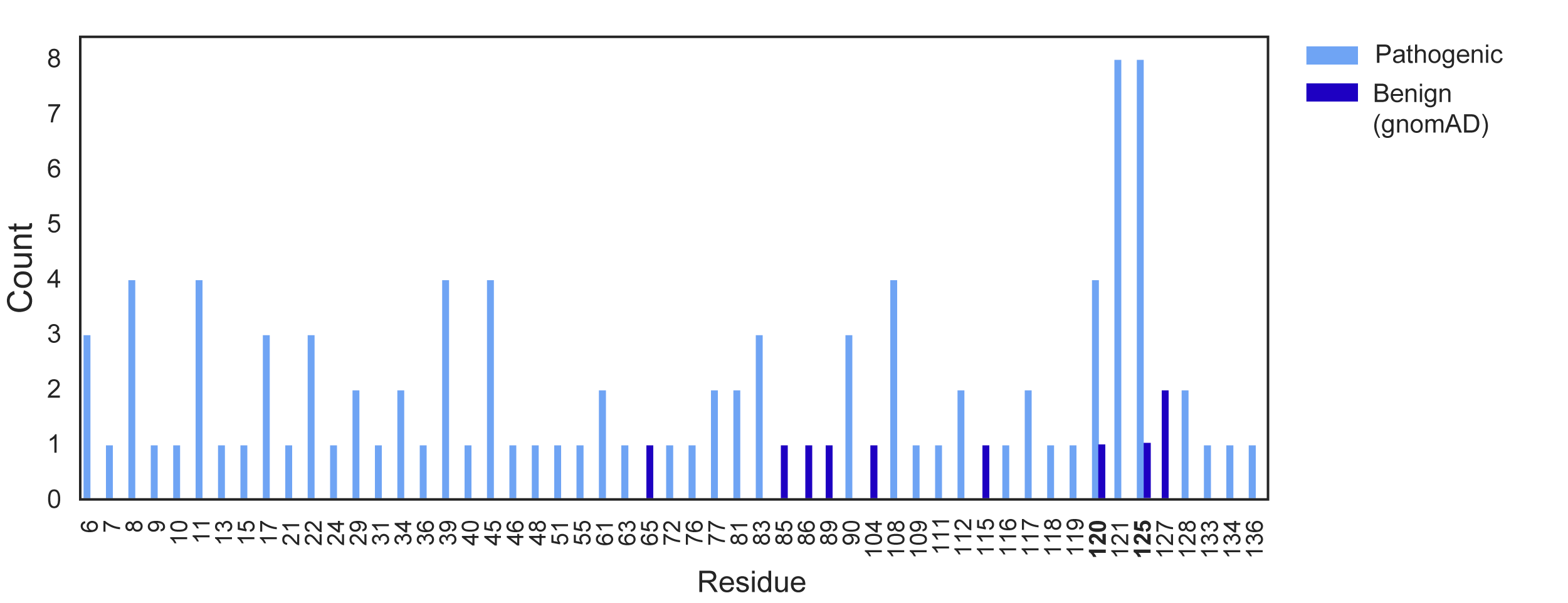

Supplement: Supplementary file 1 — Supplementary Figure 1: Observed gnomAD v2.1.1 H3-3A and H3-3B variants overlaid with BLBS variants. [file 41431_2024_1610_MOESM1_ESM.tif]
