# Supplementary material for: Expanded phenotypic spectrum of neurodevelopmental and neurodegenerative disorder Bryant-Li-Bhoj syndrome with 38 additional individuals
Source: Eur J Hum Genet. 2024 Apr 27;32(8):928–37. doi: 10.1038/s41431-024-01610-1 (PMC11291762; doi:10.1038/s41431-024-01610-1)
Supplement: Supplementary file 2 — Supplementary Figure 1: Legend [file 41431_2024_1610_MOESM2_ESM.docx]

**Supplementary Figure 1.** ***H3-3A* and *H3-3B* are intolerant to variation.** Counts of variants observed in individuals with BLBS (**blue**) or variants observed in individuals who were not ascertained for having a neurological condition in a neurological case/control study in gnomAD v2.1.1 (**purple**). Residues 120 and 125 are bolded to denote that variants at these locations are present in both individuals with BLBS and gnomAD. M120K is present in both cohorts, while individuals with BLBS have Q125R variants, while the individual in gnomAD harbors a Q125H variant.
